# The positive predictive value of MOG-IgG testing based on the 2023 diagnostic criteria for MOGAD

**DOI:** 10.1177/20552173241274610

**Published:** 2024-08-14

**Authors:** Linda Nguyen, Sumit Singh, Fabricio S Feltrin, Lauren M Tardo, Rebekah L Clarke, Cynthia X Wang, Benjamin M Greenberg

**Affiliations:** Department of Pediatrics, 12334 University of Texas Southwestern Medical Center, Dallas, TX, USA; Department of Radiology, 12334 University of Texas Southwestern Medical Center, Dallas, TX, USA; Department of Neurology, 12334 University of Texas Southwestern Medical Center, Dallas, TX, USA; Department of Radiology, 12334 University of Texas Southwestern Medical Center, Dallas, TX, USA; Department of Pediatrics, 12334 University of Texas Southwestern Medical Center, Dallas, TX, USA; Department of Neurology, 12334 University of Texas Southwestern Medical Center, Dallas, TX, USA; Department of Pediatrics, 12334 University of Texas Southwestern Medical Center, Dallas, TX, USA; Department of Neurology, 12334 University of Texas Southwestern Medical Center, Dallas, TX, USA

**Keywords:** Demyelination, MRI, MOGAD, myelin oligodendrocyte glycoprotein, positive predictive value

## Abstract

**Background:**

Myelin oligodendrocyte glycoprotein antibody associated disease (MOGAD) is a relatively new disease entity in the field of demyelinating disorders. Its first diagnostic criteria have recently been published.

**Objectives:**

We evaluated the positive predictive value (PPV) for MOG-IgG testing and report the clinical and radiologic features with respect to the recently published criteria.

**Methods:**

A retrospective study was conducted at three centers in Dallas, Texas. Patients with positive MOG-IgG testing on cell-based assays at any time were included. Positive cases were reviewed by at least two neuroimmunologists for fulfillment of the criteria.

**Results:**

We included 235 patients. The PPV of seropositivity at any time was 78.3% overall, 52.6% for low titer, and 90.1% for high titer. Children had a higher PPV than adults (93.9% versus 67.2%). Positive predictive value was 6.3% in those without a core clinical demyelinating attack. Children more often have the typical imaging features of MOGAD in optic neuritis than adults.

**Conclusions:**

We report a PPV of 78.3% for MOG-IgG testing using the 2023 MOGAD diagnostic criteria. Children had higher PPV and frequency of supporting imaging features. Careful consideration is necessary when assigning patients with no core demyelinating event and low titers a MOGAD diagnosis.

## Introduction

Myelin oligodendrocyte glycoprotein antibody associated disease (MOGAD) defines a subgroup of patients with central nervous system (CNS) inflammation with MOG-IgG that is distinct from multiple sclerosis (MS) and aquaporin-4 (AQP4)-IgG positive neuromyelitis optic spectrum disorder (NMOSD). The clinical spectrum of MOGAD is heterogenous and has evolved with increasing availability of accurate cell-based assays (CBAs) in the past decade.

The first formal, international consensus diagnostic criteria for MOGAD has recently been proposed.^1^ The key features for diagnosis require a core demyelinating clinical attack, seropositivity, and exclusion of alternative diagnoses. Furthermore, those with low positive titers require at least one supporting clinical or magnetic resonance imaging (MRI) feature while those with clear (or high) positive titers do not require additional supporting features. These criteria apply to both pediatric and adult patients.

In this study, we aimed to determine the positive predictive value (PPV) of MOG-IgG testing in a large cohort of pediatric and adult patients from three tertiary care centers in Dallas, Texas, using the recently published criteria. To reflect real-world settings, we included patients with MOG-IgG obtained at any time during the disease course. Prior studies have shown that PPV varies depending on titer level, clinical phenotype, and age of the patient.^2,3^ As such, we evaluated the PPV for different titer levels and clinical phenotypes and examined for differences in PPV between children and adults. We also reported the PPV based on titers available at initial attack or only later because the timing of testing may affect the MOG-IgG level and the diagnosis of MOGAD.^4^

Finally, MRI is a key tool in diagnosing MOGAD, as it is often needed to review for supporting features, particularly in those with low positive titers, and rule out better alternative diagnoses, regardless of titer levels. Previous studies have reported on various imaging features that may be more frequently seen or even be distinct in MOGAD compared to MS and AQP4-IgG-positive NMOSD.^1^ To add to the growing literature on this, we assessed the MRI available at first attack for the frequency of typical imaging features of MOGAD and atypical features more suggestive of MS or AQP4-IgG-positive NMOSD in the true-positive cases. We only reviewed the MRI available at first attack because 40%–60% of patients with MOGAD have a monophasic disease course^5,8,9^ and their MRI frequently shows partial or complete resolution of T2 hyperintensities after an attack.^1^

## Methods

### Patients

We queried the electronic medical records for pediatric and adult patients who were seen at Children's Medical Center, University of Texas Southwestern Medical Center or Parkland Health in Dallas, Texas, between January 2014 and April 2023 and were tested for MOG-IgG. Pediatric patients were below 18 years of age, while adult patients were 18 years old and above. Those with at least one positive MOG-IgG test at any time were further reviewed. All patients had positive serum MOG-IgG on CBAs. Additionally, positive titers were designated as “low” or “high” per the individual assay cutoffs.^1^ Specifically, for live CBAs from Mayo Clinic, low positives are 1:20–1:40 and high positives are ≥1:100. For live CBAs from the University of Oxford, low positives are scores 1–1.5 and high positive are scores ≥2. For fixed CBAs from ARUP, Athena, Labcorp, or Quest, a result at ≥1:10 is considered positive and ≥1:100 is considered high positive requiring only a core demyelinating attack. We excluded patients who had a report of positive MOG-IgG test but information on the methodology used (i.e., cell-based or not) was not available or who had insufficient clinical data for true-positive versus false-positive assessment.

### Data collection

Clinical presentation and MRI at first attack were retrospectively collected. MOG-IgG peak levels at first attack, at a later time and not at first attack, and at any time, including at onset, were also collected. The six defined core CNS clinical demyelinating events described by Banwell et al. included acute disseminated encephalomyelitis (ADEM), optic neuritis, myelitis, cerebral monofocal/polyfocal deficits, brainstem/cerebellar deficits, and cerebral cortical encephalitis often with seizures.^1^ If they presented with ADEM and other phenotypes, we grouped them in the ADEM category. If they presented with symptoms and imaging findings consistent with optic neuritis and myelitis simultaneously, we grouped them in an additional category for concurrent optic neuritis and myelitis. For all other symptoms and imaging findings not consistent with a CNS clinical demyelinating event, they were categorized as “other.”

For true-positive versus false-positive assessment, cases were reviewed for fulfillment of MOGAD criteria by treating neuroimmunologists and reviewing study authors (LN and LMT). The study authors reviewed all the medical history available, including the first attack and relapses, if the patient had any subsequent attacks. The study authors were not blinded to MOG-IgG titer level as this is a key feature of the criteria and seropositivity was required for inclusion. True-positive cases met the current international recommendations. False-positive cases were those who had an inconsistent MOGAD clinical syndrome or were felt to have a more likely alternative diagnosis. Details of the clinical and paraclinical information used to support the designation of false-positive were noted. There was consensus agreement for each case between at least two neuroimmunologists (treating neuroimmunologist and study author, or two study authors).

MRIs at first attack were reviewed for both typical features of MOGAD and atypical features more suggestive of MS or NMOSD^1^ by two pediatric neuroradiologists (SS and RLC) and one adult neuroradiologist (FSF) who were blinded to the patient's disease course. SS is the senior neuroradiologist and reviewed the first 20 scans in pediatric and adult patients with RLC and FSF, respectively, to establish interrater reliability. Any discrepancies were resolved by consensus. Pediatric patients were subsequently divided up among the two pediatric neuroradiologists and adult patients were assigned to the one adult neuroradiologist given their respective expertise in the two different patient populations, pediatric and adults. Typical features include multiple ill-defined T2 hyperintense lesions in supratentorial and often infratentorial white matter, deep gray matter involvement, perineural optic sheath enhancement, and longitudinally extensive cord lesion (eTable 1). Atypical features included Dawson's fingers, chiasmal or optic tract involvement, and multiple focal, short-segment skip cord lesions (eTable 1).

### Statistical analysis

Data were summarized with counts (percentages) for categorical variables. PPVs were calculated by dividing the true-positive results by the total positive results, with 95% confidence intervals (CIs) calculated using the modified Wald method. PPVs for the “all positive titer,” “low positive,” and “high positive” groups included patients with fixed or live CBA. PPVs for the specific titer (1:20, 1:40, 1:100, and ≥1:1000) groups included patients with only live CBA from Mayo Clinic. Children and adult groups were analyzed using the Pearson chi-squared test or Fisher's exact test for categorical variables. The analyses were performed using SPSS Statistics version 28.0 (SPSS, Inc., Chicago, IL) and GraphPad Prism version 9.5 (GraphPad Software, La Jolla, CA). *P* < 0.05 (two-sided) was considered significant.

### Standard protocol approvals, registrations, and patient consents

This study was approved by the Institutional Review Board of the University of Texas Southwestern Medical Center. Written informed consent for publication was waived due to the retrospective nature of the study.

### Data availability

Anonymized data not published within this article will be made available by request from any qualified investigator.

## Results

**Figure 1.**
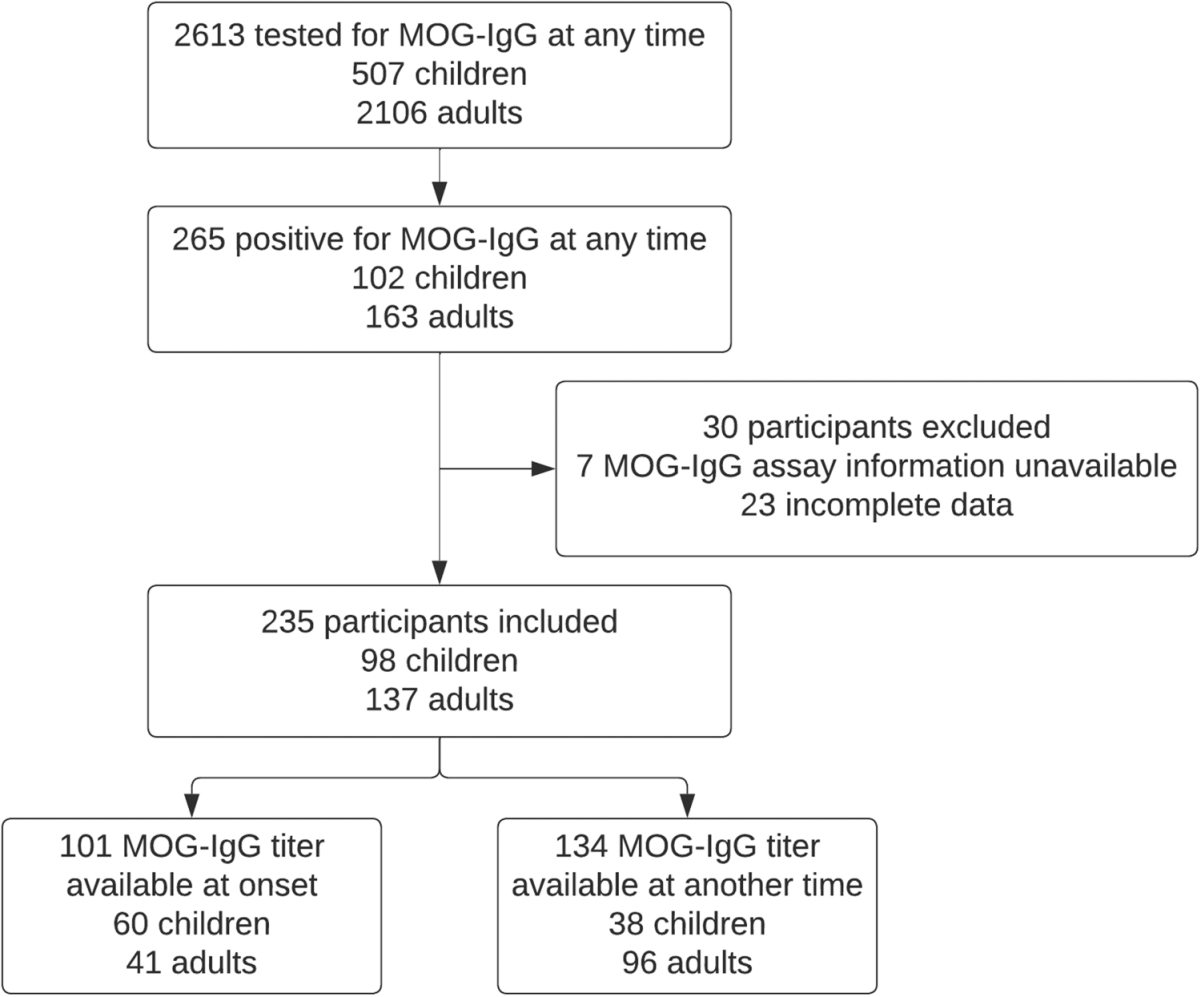
Flowchart of patient selection with MOG-IgG testing for fulfillment of myelin oligodendrocyte glycoprotein antibody associated disease (MOGAD) criteria.

Of the 2613 patients who were tested for MOG-IgG, 265 patients had at least one positive test (Figure 1). The prevalence of a positive MOG-IgG test was higher in children than adults (20.1% versus 7.7% in adults, *p* < 0.001). Thirty positive cases were excluded because assay information was not available (*n* = 7) or patient had incomplete clinical and MRI data (*n* = 23). A total of 235 patients were further reviewed for fulfillment of the MOGAD diagnostic criteria: 102 had a positive CBA test at initial attack (101 of these had titers available) and 133 had a positive CBA test only after initial attack (130 of these had titers available and three initially had negative testing at initial attack). Of the 235 patients, 231 had at least one positive test via a live CBA. The other four had seropositivity based on only fixed CBA (one with titer result not available, two with titers of 1:10, and one with titer of 1:160). One patient with positive MOG-IgG was also positive for AQP4-IgG. Clinical phenotypes at onset were 17.4% ADEM, 44.3% optic neuritis, 12.8% myelitis, 6.4% cerebral monofocal/polyfocal deficits, 2.6% brainstem/cerebellar deficits, 1.7% cerebral cortical encephalitis, 3.8% concurrent optic neuritis and myelitis, and 6.8% other. Ten (4.3%) patients had unknown/insufficient data on onset symptoms. “Other” presentations included five with nonspecific neurologic symptoms or symptoms lasting <24 h, one with seizure and normal brain MRI, three with progressive symptoms over years, one with acute demyelinating polyneuropathy, one with retinitis, one with presumed infectious meningoencephalitis, one with worsening mental status and ischemic brain lesions in the setting of critical illness, one with failed vision screen, one with unilateral temporal hemianopsia with negative MRI and optical coherence tomography, and one with concussion.

When examining those with fixed or live CBA, the PPV based on peak seropositivity at any time was 78.3% (95% CI, 72.6–83.1%) overall, 52.6% (95% CI, 41.6–63.3%) for low titer, and 90.1% (95% CI, 84.0–94.1%) for high titer (Table 1). A fixed CBA titer was used to calculate these PPVs in five patients (two had titers of 1:10, one had titer of 1:2560, one had a titer of 1:160, and one had no titer available). To determine if a higher cutoff would change PPV, we examined only those with peak titer at any time available through the Mayo Clinic. With a cutoff of 1:20, the PPV was 77.9% (173/222; 95% CI, 72.0–82.9%). With a cutoff of 1:40, the PPV increased to 85.6% (155/181; 95% CI, 79.7–90.1%). Evaluating whether the timing of MOG-IgG collection (at initial attack or at a later time) affected the PPV, we found that the PPV based on seropositivity at onset was 87.3% (95% CI, 79.3–92.5%) overall, 60.7% (95% CI, 42.4–76.5%) for low titer, and 97.3% (95% CI, 90.0–99.8%) for high titer. The PPV based on seropositivity only later and not at onset was 71.4% (95% CI, 63.2–78.4%) overall, 52.6% (95% CI, 39.9–65.0%) for low titer, and 83.6% (95% CI, 73.3–90.5%) for high titer. PPV was significantly higher for those with titers available at initial attack versus only later for overall and high titers (*p* = 0.003 and *p* = 0.005, respectively), but not for low titers (*p* = 0.481).

**Table 1.** Positive predictive value for patients with positive MOG-IgG testing.

	Total cohort No (%) [95% CI]	Children No (%) [95% CI]	Adult No (%) [95% CI]	*p*-value
Peak titer available at any time, including onset				
All positive titer	184/235 (78.3) [72.6, 83.1]	92/98 (93.9) [87.0, 97.4]	92/137 (67.2) [58.9, 74.5]	<0.001
Low positive	41/78 (52.6) [41.6, 63.3]	18/23 (78.3) [57.7, 90.8]	23/55 (41.8) [29.7, 55.0]	0.003
1:20	18/41 (43.9) [29.9, 59.0]	6/10 (60.0) [31.2, 83.3]	12/31 (38.7) [23.7, 56.2]	0.238
1:40	19/31 (61.3) [43.8, 76.3]	8/9 (88.9) [54.3, 100.0]	11/22 (50.0) [30.7, 69.3]	0.044
High positive	128/142 (90.1) [84.0, 94.1]	62/63 (98.4) [90.7, 100.0]	66/79 (83.5) [73.7, 90.3]	0.003
1:100	84/98 (85.7) [77.3, 91.4]	41/42 (97.6) [86.6, 100.0]	43/56 (76.8) [64.1, 86.0]	0.004
≥1:1000	52/52 (100.0) [91.8, 100.0]	29/29 (100.0) [86.1, 100.0]	23/23 (100.0) [83.1, 100.0]	–
Titer available at onset				
All positive titer	89/102 (87.3) [79.3, 92.5]	57/60 (95.0) [85.8, 98.8]	32/42 (76.2) [61.3, 86.7]	0.005
Low positive	17/28 (60.7) [42.4, 76.5]	9/12 (75.0) [46.2, 91.7]	8/16 (50.0) [28.0, 72.0]	0.180
1:20	7/15 (46.7) [24.8, 69.9]	3/5 (60.0) [22.9, 88.4]	4/10 (40.0) [16.7, 68.8]	0.608
1:40	10/12 (83.3) [54.0, 96.5]	6/7 (85.7) [46.7, 99.5]	4/5 (80.0) [36.0, 98.0]	1.000
High positive	71/73 (97.3) [90.0, 99.8]	48/48 (100.0) [91.2, 100.0]	23/25 (92.0) [73.9, 98.9]	0.114
1:100	34/36 (94.4) [80.9, 99.4]	23/23 (100.0) [83.1, 100.0]	11/13 (84.6) [56.5, 96.9]	0.124
≥1:1000	36/36 (100.0) [88.5, 100.0]	25/25 (100.0) [84.2, 100.0]	11/11 (100.0) [70.0, 100.0]	–
Peak titer available at later time and not at onset				
All positive titer	95/133 (71.4) [63.2, 78.4]	35/38 (92.1) [78.5, 98.0]	60/95 (63.2) [53.1, 72.2]	0.001
Low positive	30/57 (52.6) [39.9, 65.0]	12/14 (85.7) [58.8, 97.2]	18/43 (41.9) [28.4, 56.7]	0.004
1:20	12/29 (41.4) [25.5, 59.3]	3/5 (60.0) [22.9, 88.4]	9/24 (37.5) [21.1, 57.4]	0.622
1:40	14/23 (60.9) [40.7, 77.9]	5/5 (100.0) [51.1, 100.0]	9/18 (50.0) [29.0, 71.0]	0.116
High positive	61/73 (83.6) [73.3, 90.5]	21/22 (95.5) [76.5, 100.0]	40/51 (78.4) [65.2, 87.7]	0.072
1:100	46/58 (79.3) [67.1, 87.9]	16/17 (94.1) [71.1, 100.0]	30/41 (73.2) [57.9, 84.4]	0.073
≥1:1000	13/13 (100.0) [73.4, 100.0]	3/3 (100.0) [38.3, 100.0]	10/10 (100.0) [67.9, 100.0]	–

PPV based on clinical phenotype at onset ranged from 50.0% to 100.0% for those with a core clinical demyelinating event (Table 2). In those with no core clinical demyelinating event (i.e., other presentation), the PPV was 6.3% (95% CI, 0.0–30.3%). Comparing children versus adults, based on peak seropositivity at any time, children had higher overall PPV than adults (93.9% [95% CI, 87.0–97.4%] versus 67.2% [95% CI, 58.9–74.5%]). Similarly, based on seropositivity at onset or a later time, children had higher overall PPV than adults (95.0% [95% CI, 85.8–98.8%] versus 76.2% [95% CI, 61.3–86.7%] and 92.1% [95% CI, 78.5–98.0%] versus 63.2% [95% CI, 53.1–72.2%], respectively).

**Table 2.** Positive predictive value for patients with positive MOG-IgG testing based on clinical phenotype at onset.

	Total cohort No (%) [95% CI]	Children No (%) [95% CI]	Adult No (%) [95% CI]	*p*-value
Acute disseminated encephalomyelitis	40/42 (95.2) [83.4, 99.5]	37/38 (97.4) [85.3, 100.0]	3/4 (75.0) [28.9, 96.6]	0.184
Optic neuritis	90/104 (86.5) [78.5, 91.9]	32/35 (91.4) [76.9, 97.8]	58/69 (84.1) [73.5, 91.0]	0.298
Myelitis	22/32 (68.8) [51.3, 82.2]	9/9 (100.0) [65.5, 100.0]	13/23 (56.6) [36.8, 74.4]	0.030
Cerebral monofocal/polyfocal deficits	12/15 (80.0) [54.1, 93.7]	8/9 (88.9) [54.3, 100.0]	4/6 (66.7) [29.6, 90.8]	0.525
Brainstem/cerebellar deficits	3/6 (50.0) [18.8, 81.2]	2/3 (66.7) [20.2, 94.4]	1/3 (33.3) [5.6, 79.8]	1.000
Cerebral cortical encephalitis	4/4 (100.0) [45.4, 100.0]	3/3 (100.0) [38.3, 100.0]	1/1 (100.0) [16.8, 100.0]	1.000
Concurrent optic neuritis and myelitis	8/8 (100.0) [62.8, 100.0]	–	8/8 (100.0) [62.8, 100.0]	–
Other	1/16 (6.3) [0.0, 30.3]	1/2 (50.0) [9.5, 90.6]	0/14 (0.0) [0.0, 25.2]	0.125

Examining a subgroup of 52 patients who had positive MOG-IgG at first attack and follow-up for at least a year, 47 met the diagnostic criteria for MOGAD at first attack. None had a change in their diagnosis on long-term follow-up. Of the three patients who had negative MOG-IgG on CBA at onset but later converted to positive, two subsequently fulfilled the consensus criteria. One was an adult patient with left optic neuritis and was treated with steroids (initially low-dose oral steroids, then high-dose intravenous steroids followed by oral steroid taper) starting 20–30 days prior to MOG-IgG collection and the other was a pediatric patient with ADEM who received a course of high-dose intravenous steroids followed by short oral steroid taper starting three weeks prior to MOG-IgG collection.

A total of 51 patients (21.7%) (6 children, 45 adults) had their results designated as false positive (Table 3). Of the 51 patients, 29 (56.9%) had an alternate diagnosis of MS. Additional details of each false-positive case are provided in the supplemental materials.

**Table 3.** Clinical diagnoses and corresponding serum MOG-IgG titers for false-positive cases.

Patient number	Age group	Clinical diagnosis	Serum MOG-IgG titer at onset	Peak MOG-IgG titer at any time
1	Children	Multiple sclerosis, *n* = 4	1:20	1:20
2			1:20	1:20
3			N/A	1:20
4			N/A	1:20
5		Genetically confirmed hemophagocytic lymphohistiocytosis, n = 1	Negative	1:100
6		Other non-neuroimmunologic condition, *n* = 1	1:40	1:40
7	Adult	Multiple sclerosis, *n* = 25	1:10 (ARUP)	1:10 (ARUP)
8			1:20	1:20
9			N/A	1:10 (Quest)
10			N/A	1:20
11			N/A	1:20
12			N/A	1:20
13			N/A	1:20
14			N/A	1:20
15			N/A	1:20
16			N/A	1:20
17			N/A	1:20
18			N/A	1:20
19			N/A	1:40
20			N/A	1:40
21			N/A	1:40
22			N/A	1:40
23			N/A	1:40
24			N/A	1:40
25			N/A	1:100
26			N/A	1:100
27			N/A	1:100
28			N/A	1:100
29			N/A	1:100
30			N/A	1:100
31			N/A	1:100
32		Aquaporin-4-IgG-positive neuromyelitis optica, *n* = 1	N/A	1:20
33		Possible/probable neurosarcoidosis, *n* = 2	N/A	1:100
34			N/A	1:100
35		Other transverse myelitis, *n* = 1	N/A	1:40
36		Other optic neuritis, *n* = 1	N/A	1:40
37		Other neuroimmunologic condition, *n* = 3	1:40	1:40
38			1:100	1:100
39			N/A	1:20
40		Unspecified neuroinflammatory condition, *n* = 1	1:20	1:20
41		Ischemic optic neuropathy, *n* = 1	1:20	1:20
42		Other optic neuropathy, *n* = 1	N/A	1:20
43		Embolic strokes, *n* = 1	1:100	1:100
44		Acute inflammatory demyelination polyneuropathy, *n* = 1	1:20	1:20
45		HIV retinopathy, *n* = 1	1:20	1:20
46		Presumed infectious meningoencephalitis, *n* = 1	N/A	1:40
47		Functional neurological disorder, *n* = 1	N/A	1:20
48		Other non-neuroimmunologic condition, *n* = 3	1:20	1:40
49			N/A	1:20
50			N/A	1:100
51			N/A	1:100

MOG-IgG assays were performed at Mayo clinic laboratories unless otherwise noted.

Among the true-positive cases, clinical phenotypes at onset were 21.7% ADEM (40.2% in children, 3.3% in adults), 48.9% optic neuritis (34.8% in children, 63.0% in adults), 12.0% myelitis (9.8% in children, 14.1% in adults), 6.5% cerebral monofocal/polyfocal deficits (8.7% in children, 4.3% in adults), 1.6% brainstem/cerebellar deficits (2.1% in children, 1.1% in adults), 2.2% cerebral cortical encephalitis (3.3% in children, 1.1% in adults), 4.3% concurrent optic neuritis and myelitis (0.0% in children, 8.7% in adults), and 0.5% other (1.1% in children, 0.0% in adults). Four (2.2%) patients had unknown/insufficient data on onset symptoms. Of those presenting with optic neuritis with/without myelitis and known laterality, 32.3% (42.4% [14/33] in children, and 27.3% [18/66] in adults) had bilateral vision changes. In those with imaging at onset available for review, bilateral involvement of the optic nerve (either clinically and/or radiologically) was present in 45.5% (53.1% [17/32] in children, and 40.0% [18/45] in adults]. On fundoscopic exam, 83.3% (81.3% [26/32] in children, 84.6% [33/39] in adults) had optic nerve head edema. The “other” presentation in one child was headaches only with multifocal supratentorial white matter lesions, deep gray matter involvement, cortical lesions, and meningeal enhancement.

**Figure 2.**
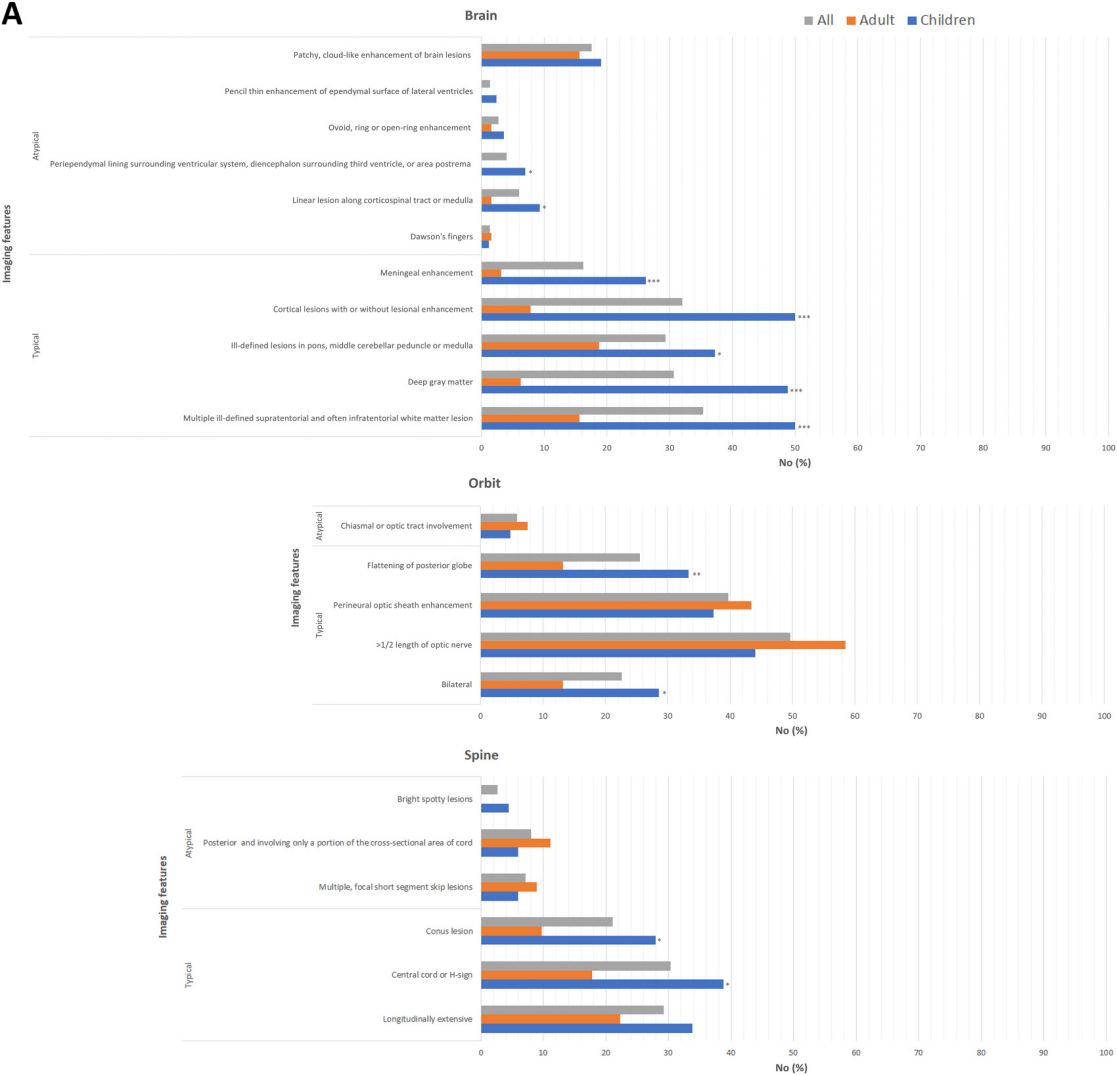

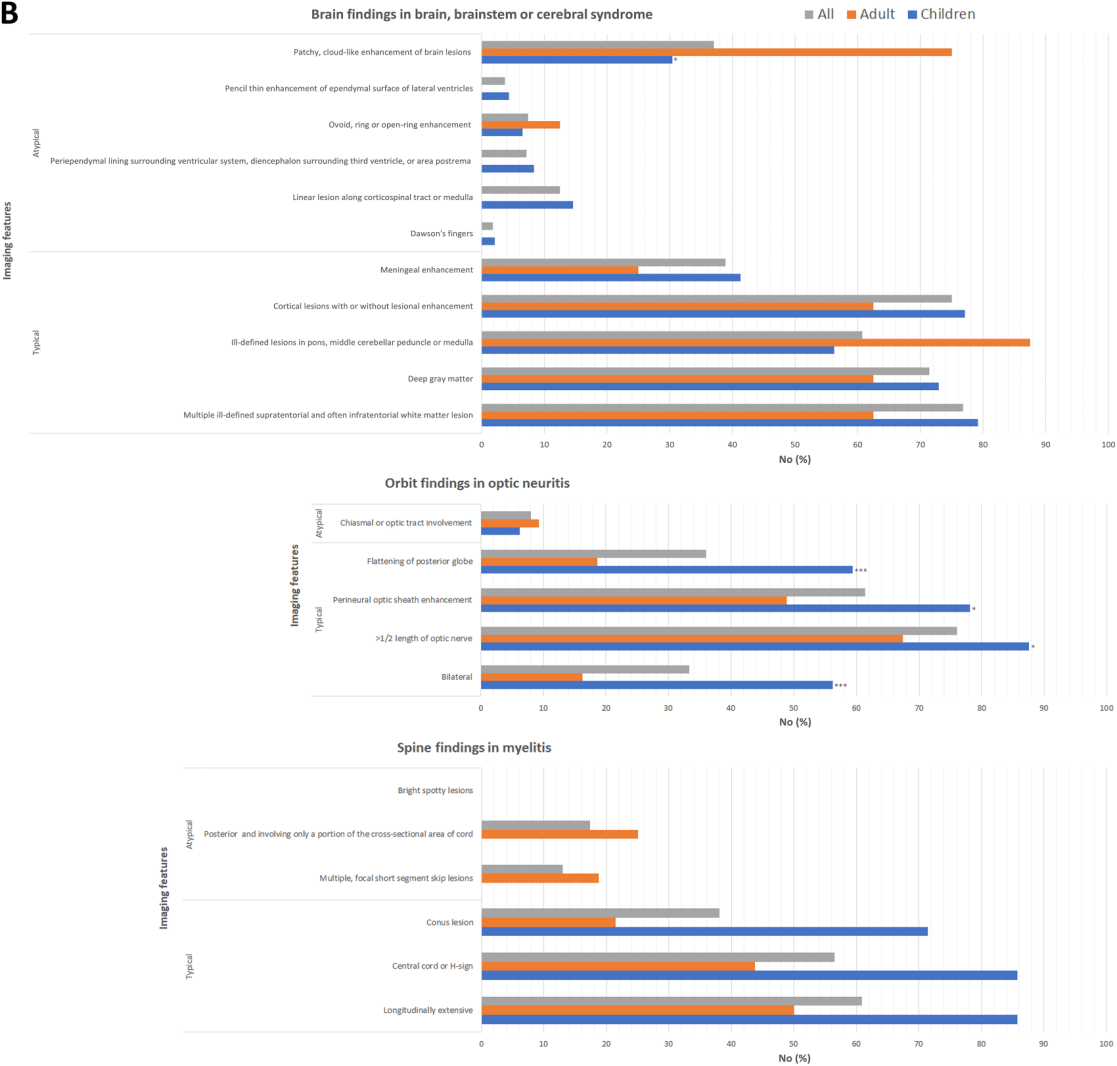
Magnetic resonance imaging (MRI) features based on imaging location (A) or clinical syndrome (B). *p < 0.05; **p < 0.01; ***p < 0.001 children versus adult.

On combined review of the first 20 cases to establish interrater reliability between two neuroradiologists, there were discrepancies in 8/460 variables reviewed for the pediatric cases and 34/460 for the adult cases. The discrepancies were resolved by consensus. The rates of typical and atypical MRI features for true-positive cases based on imaging location or clinical presentations are shown in Figure 2 and eTables 2 and 3. The most frequent atypical feature was patchy, cloud-like enhancement of brain lesions, which was seen in 17.6% of cases (19.0% in children, 15.6% in adults) based on imaging location and 37.0% of cases (30.4% in children and 75.0% in adults) based on clinical presentation. In those presenting with symptomatic optic neuritis, children more often had typical imaging features of bilateral involvement (53.6% versus 16.3%, *p* < 0.001), >1/2 length of optic nerve (87.5% versus 67.4%, *p* = 0.44), perineuritis (78.1% versus 48.9%, *p* = 0.010), and flattening of the posterior globe (59.4% versus 18.6%, *p* ≤ 0.001).

## Discussion

The present study determined the PPV for MOG-IgG testing based the recent consensus criteria for MOGAD in a broad, real-world population. Our study cohort is the largest to date evaluating the PPV of MOG-IgG testing in clinical practice. We report the following important observations: (1) increasing PPV with higher titers; (2) children had a higher PPV than adults; and (3) very low PPV in those who do not present with a core clinical demyelinating attack. These findings reiterate that PPV is dependent on the selection of individuals and disease prevalence within a given population.

Our PPVs are similar to previous studies. Sechi et al. had shown a PPV of 71.7% (66/92) overall, 51.2% (21/41) with titers 1:20–1:40, 81.8% (27/33) with titer 1:100, and 100.0% (17/17) with titers ≥1:1000. Manzano et al. had shown a PPV of 85.9% (67/78) with titer cutoff of 1:20 and 92.3% (60/65) with titer cutoff of 1:40.^10^ In our cohort, PPV was 78.3% (184/235) with any positive CBA (fixed or live and performed by any lab). PPV was 77.9% (173/222) and 85.6% (155/181) with titer cutoff of 1:20 and 1:40 (from only live CBA performed by Mayo Clinic), respectively. Moreover, Sechi et al. showed children had higher PPV compared to adults (93.8% [15/16] versus 67.1% [51/76]) and very low PPV in those with atypical presentations (11.8% [2/17]). We also report that while increasing the cutoff to 1:40 increases the PPV, a relatively significant number of patients (18/222, or 8.1%), still fit the diagnosis of MOGAD at titers of <1:40. Thus, excluding patients with positive titers <1:40 without proper clinical analysis may lead to a significant number of missed MOGAD diagnoses.^2, 10^

Examining MRI features in true-positive cases only, we found the frequencies for the typical features based on clinical presentation ranged from 33.3% to 78.2% and are, for the most part, consistent with relative frequencies previously reported.^1^ Except for patchy, cloud-like enhancement of brain lesions, all atypical features were infrequent, occurring <20% of the time. This suggests that the patchy, cloud-like lesion enhancement pattern may not be unique to AQP4-IgG-positive NMOSD,^11^ which warrants confirmation in future studies that will include both MOGAD and AQP4-IgG-positive NMOSD. Interestingly, we found children more often demonstrated the typical supporting imaging features for MOG-IgG associated optic neuritis than adults. Notably, adults infrequently (<20%) had bilateral optic nerve involvement and flattening of posterior globe on neuroimaging compared to children. These radiologic differences suggest different pathophysiologic mechanisms in the anterior visual pathway and should be confirmed in larger, multicenter cohorts.

Our study has limitations. First, it is retrospective by design and enrolled patients from a single geographic location. Second, our primary outcome was PPV based on peak seropositivity at any time. While this better represents real-world settings and increases the study's generalizability, using seropositivity at any timepoint during the patient's disease course increases heterogeneity. Notably, we found the PPV was significantly higher for those who had positive titers available at onset versus only later. Also, a relatively large proportion of our cohort (28.9%, or 68/235) did not/have not had follow-up for at least a year. It is possible that the true-positive and false-positive cases may change with longer follow-up. We found, however, in a subset of 52 patients with positive MOG-IgG at first event and follow-up for at least a year, none had a change in their diagnosis from first event to long-term follow-up, suggesting that the current diagnostic criteria is highly accurate. Nevertheless, future studies are needed to establish whether individuals who have false-positive MOG-IgG, especially those with high titers, develop clinical MOGAD attacks at a later point. Finally, the fulfillment of MOGAD criteria requires the exclusion of better diagnoses. It is possible that an overlap CNS immune-mediated syndrome may exist (e.g., *N*-methyl-d-aspartate receptor encephalitis-MOGAD^12^), which requires exploration in future studies.

## Conclusion

We investigated the PPV of MOG-IgG testing considering the recently proposed 2023 international consensus criteria for the diagnosis of MOGAD. We found a false-positive rate of 21.7%. Caution is advised in interpreting positive MOG-IgG results in patients with no core clinical attack phenotype and titers <1:40, especially in adults. Therefore, thoughtful clinical judgment with comprehensive understanding of the clinical and radiologic characteristics of MOGAD remains necessary to maximize the usefulness of the MOG-IgG test with the 2023 criteria.^13^

## Supplemental Material

sj-docx-1-mso-10.1177_20552173241274610 - Supplemental material for The positive predictive value of MOG-IgG testing based on the 2023 diagnostic criteria for MOGAD Supplemental material, sj-docx-1-mso-10.1177_20552173241274610 for The positive predictive value of MOG-IgG testing based on the 2023 diagnostic criteria for MOGAD by Linda Nguyen, Sumit Singh, Fabricio S Feltrin, Lauren M Tardo, Rebekah L Clarke, Cynthia X Wang and Benjamin M Greenberg in Multiple Sclerosis Journal – Experimental, Translational and Clinical
