# Supplementary material for: The positive predictive value of MOG-IgG testing based on the 2023 diagnostic criteria for MOGAD
Source: Mult Scler J Exp Transl Clin. 2024 Aug 14;10(3):20552173241274610. doi: 10.1177/20552173241274610 (PMC11325327; doi:10.1177/20552173241274610)
Supplement: sj-docx-1-mso-10.1177_20552173241274610 - Supplemental material for The positive predictive value of MOG-IgG testing based on the 2023 diagnostic criteria for MOGAD [file sj-docx-1-mso-10.1177_20552173241274610.docx]

**Supplemental materials**

Rationale for false-positive designation.

Patient 1: Patient presented with headaches and vision loss in right eye. MRI orbit showed long segment signal changes with enhancement of the right optic nerve, along with perineural optic nerve sheath enhancement and flattening of the posterior globe. MRI brain showed T2 hyperintensities in the bilateral subcortical, deep and periventricular white matter (with Dawson’s fingers appearance) without enhancement. MRI spine normal. CSF analysis showed 7 nucleated cells (74% lymphocytes), elevated protein, 2 oligoclonal bands, and elevated IGG index 0.74. Fundoscopic exam showed optic nerve head edema in the right eye. Serial MRI brain outpatient showed unchanged T2 signal changes.

Patient 2: Patient presented with left eye pain and blurred vision. MRI orbit showed short segment signal changes in the left optic nerve with enhancement. MRI brain showed Dawson’s fingers and other T2 hyperintensities in the centrum semiovale, corona radiata and pons, with ring/open-ring enhancement. MRI spine negative. CSF analysis showed normal nucleated cells and protein, 15 oligoclonal bands, and elevated IgG index 0.93. Fundoscopic exam without optic nerve head edema.

Patient 3: Patient presented with right-sided weakness and numbness. MRI brain showed left midbrain lesion. CSF analysis showed normal nucleated cells and 5 oligoclonal bands. Repeat MRI brain four months later showed new left parietal lesion.

Patient 4: Patient presented with left optic neuritis. MRI brain showed enhancing lesion in the left optic nerve and non-enhancing ovoid lesions in the subcortical and callosal regions and left cerebellar peduncle. MRI 4 months later showed new ovoid lesions in the bilateral frontal lobe. MRI spine negative. No CSF studies. OCT with thinning in the retinal nerve fiber layer, predominately in the temporal quadrant, on the left.

Patient 5: Patient presented with headaches for 3 weeks. MRI brain showed numerous supratentorial and infratentorial lesions with enhancement and restricted diffusion. The supratentorial lesions predominately involved the cortex and basal ganglia. There were multiple foci of hemorrhage. MRI spine showed enhancing, expansile, central cord, longitudinally extensive lesions. CSF analysis showed 9 nucleated cells, normal protein, negative oligoclonal bands, and elevated IgG index 0.97. Brain biopsy showed findings consistent with meningoencephalitis characterized by foci of lymphohistiocytic perivascular inflammation. There were features suggestive of, but not definitive for demyelination. Subsequently due to a family presenting with similar CNS syndrome, the patient underwent genetic testing and was found to have a homozygous mutation in PRF1 gene and functional assays showed nearly complete absent PRF activity.

Patient 6: Patient presented with seizure-like activity and worsening headaches. MRI brain negative. CSF analysis with normal nucleated cells and protein, negative oligoclonal bands and normal IgG index. Repeat MOG-IgG testing 2 weeks later was negative without any empiric immunotherapy.

Patient 7: Patient presented with headaches, right eye vision loss, and unsteady gait. MRI orbit showed short segment signal changes in the right prechiasmatic optic nerve and chiasm/optic tract. MRI brain showed T2 hyperintensities in the supratentorial and infratentorial regions with Dawson’s fingers. MRI spine showed multifocal, short segment lesions in the cervical and thoracic regions with patchy enhancement. CSF analysis showed 8 nucleated cells (100% lymphocytes), normal protein, and 14 oligoclonal bands.

Patient 8: Patient presented with progressive gait instability and balance issues for 1 year. MRI brain showed Dawson's fingers and MRI spine showed posteriorly oriented, multifocal short segment lesions. CSF analysis was not done.

Patient 9: Patient presented with left eye vision loss and pain. MRI orbit showed long segment left optic nerve lesion with enhancement. MRI brain showed Dawson’s fingers and juxtacortical lesions. CSF analysis was not done.

Patient 10: Patient developed blurry vision in her right eye and after a few days also her left eye. MRI brain showed multiple areas of increased ovoid T2 signal within the periventricular and subcortical white matter along with a prominent left frontal juxtacortical and right posterior corpus callosum lesion, some with enhancement. CSF analysis showed 30 nucleated cells (94% lymphocytes), 6 unique oligoclonal bands and IgG index 2.07. Optical coherence tomography showed retinal nerve fiber layer thickness of 93 on the right eye and 87 on the left eye five years after the initial attack.

Patient 11: Patient presented with sensory changes in her left chest and legs. MRI brain showed multifocal ovoid to ill-defined hyperintensities in the periventricular and subcortical white matter, some with ring/open-ring enhancement. MRI spine showed a single, short segment, central cord, enhancing lesion in the upper thoracic region. CSF analysis was negative for nucleated cells, oligoclonal bands and IgG index.

Patient 12: Patient presented with left eye vison loss and pain. MRI orbit showed increased signal and enhancement of the left optic nerve, MRI brain showed Dawson’s fingers, and MRI spine showed multifocal, posteriorly oriented, short segment lesions. CSF analysis showed positive oligoclonal bands (exact value not known) and IgG index 1.25.

Patient 13: Patient developed sensory changes in legs. MRI showed a short segment cervical cord lesion. Later developed weakness and sensory changes in hands, repeat MRI with enlarging lesions in brain and spine with enhancement. No CSF studies.

Patient 14: Patient initially with double vision, then later developed leg weakness. MRI brain over time with juxtacortical, periventricular, pericallosal and infratentorial lesions. MRI spine with short segment cervical and thoracic cord lesions. CSF analysis showed 9 nucleated cells (96% lymphocytes), 18 oligoclonal bands, and IgG index 13.2 and synthetic rate 39.4.

Patient 15: Patient developed headache and later gait difficulties and left sided paresthesia. MRI brain over time with new lesions, including in periventricular and infratentorial region. CSF analysis with greater than 5 oligoclonal bands.

Patient 16: Patient with history of lower extremity sensory changes and gait difficulty. MRI brain with enhancing and non-enhancing lesions in periventricular region and middle cerebellar peduncle. MRI spine with multifocal short segment lesions. No CSF studies.

Patient 17: Patient initially with gait impairment. MRI brain over time with enhancing and non-enhancing periventricular and juxtacortical lesions as well as global atrophy. No CSF studies.

Patient 18: Patient with initially a constellation of symptoms, including vertigo, slurred sleep, diplopia, and right arm and leg weakness. MRI brain over time with lesions in pericallosal and juxtacortical/subcortical regions. No CSF studies.

Patient 19: Patient presented with sensory changes. MRI brain over time with multiple enhancing and non-enhancing supratentorial and infratentorial lesions including in the periventricular region with Dawson’s finger appearance. MRI spine with short segment cervical cord lesions. No CSF studies.

Patient 20: Patient presented with left-sided blurry vision, vertigo and difficulty speaking. MRI brain over time with multiple enhancing and non-enhancing supratentorial and infratentorial lesions, including lesions in periventricular region with Dawson’s finger appearance. MRI spine negative. No CSF studies.

Patient 21: Patient initially with right optic neuritis. MRI brain over time with periventricular, juxtacortical, pericallosal and infratentorial lesions. MRI spine with multifocal short segment cord lesions. No CSF studies.

Patient 22: Patient initially with left optic neuritis. MRI brain over time with periventricular, pericallosal and infratentorial lesions. MRI spine with multifocal short segment cord lesions. No CSF studies.

Patient 23. Patient initially with diplopia and left facial weakness. MRI brain over time with periventricular and left cerebellar hemisphere lesions. MRI spine with short segment cervical cord lesions. CSF analysis with 4 oligoclonal bands.

Patient 24: Patient initially with bilateral lower and upper extremity sensory changes. MRI brain with enhancing and non-enhancing lesions in periventricular, juxtacortical/subcortical and infratentorial regions. MRI spine with multifocal short segment lesions. CSF analysis with greater than 5 oligoclonal bands.

Patient 25: Patient presented with unilateral vision loss with pain. MRI brain showed short segment left optic nerve hyperintensity and several T2 lesions supratentorially, including one in the body of the corpus callosum. Subsequent MRIs showed additional supratentorial lesions, including one in left temporal periventricular lesion, and scattered foci of short segment T2 hyperintensities in cervical cord. CSF analysis was not done.

Patient 26: Patient presented left sided weakness. MRI brain with periventricular and juxtacortical lesions. MRI spine with a short segment thoracic cord lesion. No CSF studies.

Patient 27: Patient developed left foot drag followed by bilateral hand numbness and pain in thoracic area. MRI brain over time with lesions in supratentorial regions, predominantly periventricular with Dawson’s fingers appearance. MRI spine with multifocal short segment lesions in cervical and thoracic region. No CSF studies.

Patient 28: Patient developed dizziness and sensory changes following childbirth. MRI brain with Dawson’s finger appearance. Serial MRI with new lesion. No CSF studies.

Patient 29: Patient initially with right optic neuritis. MRI brain over time with periventricular and subcortical/juxtacortical lesions. MRI spine with multiple short segment lesions. CSF studies not available.

Patient 30: Patient with at least two clinical attacks. MRI brain with periventricular lesions and black holes. MRI spine with lesion in the upper cervical spine spanning almost 3 segments. CSF studies not available.

Patient 31: Patient with history of paresthesia and diplopia. MRI brain over time with periventricular, subcortical/juxtacortical, pericallosal and infratentorial lesions as well as progressive brain atrophy. MRI spine with multifocal short segment lesions. No CSF studies.

Patient 32: Patient initially with right optic neuritis and later developed left optic neuritis followed by transverse myelitis. She was found to have positive aquaporin-4 antibodies 10 years after disease onset, and concurrent aquaporin-4 antibodies (1:10000) and MOG antibodies (1:20) three years later.

Patient 33: Patient presented with subacute right arm paresthesia associated with gait dysfunction, followed by left arm paresthesia. MRI brain was negative. MRI spine showed longitudinally extensive cord signal changes with mostly dorsal enhancement. CSF analysis showed 9 nucleated cells (83% lymphocytes), elevated protein and elevated glucose. Oligoclonal bands were negative. He later developed progressive neuropathic pain. MRI spine shows persistent enhancement 12 months from disease onset. He does not have pathologic confirmation of granulomatous disease.

Patient 34: Patient presented with ascending bilateral extremity numbness. MRI brain was negative. MRI spine showed longitudinally extensive lesion in the thoracic region, with trident sign. CSF analysis showed 28 nucleated cells (92% lymphocytes), elevated protein and elevated glucose. Oligoclonal bands and IgG index were not done. Repeat MRI 6 months showed persistent enhancement. He had pathologic confirmation of systemic granulomatous disease.

Patient 35: Patient presented with subacute bilateral lower extremity sensory disturbances. MRI brain was normal. MRI spine showed posteriorly oriented lesion at T10. CSF analysis was not available.

Patient 36: Patient presented with headaches and decreased peripheral vision and eye pain in the right eye. Fundoscopic exam without optic disc edema. MRI orbit with no definite abnormal signal or enhancement in the optic nerves. MRI brain and spine negative. CSF analysis showed normal nucleated cells and protein, negative oligoclonal bands and IgG index. OCT with mild retinal nerve fiber layer (RNFL) thinning over time in the left eye (117 and 107 µm at 1 month and 3.5 months after initial attack, respectively).

Patient 37: Patient with subacute bilateral lower extremity weakness and bowel/bladder dysfunction. MRI brain with non-specific white matter changes. MRI spine with nodular intramedullary enhancing lesions associated with abnormal cord signal within the lower thoracic spinal cord/conus. CSF analysis with 10 nucleated cells (98% lymphocytes), normal protein, and 3 oligoclonal bands. CSF cytology negative, while flow cytometry showed a minute population of polytypic B lymphocytes demonstrating a marked lambda light chain excess, of which the significance is uncertain.

Patient 38: Patient developed progressive lower extremity weakness over 5+ years. Exam with increased tone in upper and lower extremities with hyperreflexia, however there was also fasciculations throughout and significant atrophy in the upper and lower extremities. MRI brain with a few periventricular and deep white matter hyperintensities and MRI spine with patchy short segment hyperintensities in cervical and thoracic region. CSF analysis was not done.

Patient 39: Patient with history of suspected left sided uveitis 5 years prior who developed severe retro-orbital eye pain followed by pain with movement and blurry vision. Fundoscopic exam revealed left optic nerve pallor. MRI orbit showed left optic nerve atrophy and short segment signal changes without enhancement. MRI brain and spine were negative. She was treated with steroids which improved the pain but not the vision.

Patient 40: Patient initially admitted for weakness and confusion. Hospital course complicated by seizures, pneumonia and respiratory failure requiring intubation. MRI brain with multifocal enhancing and non-enhancing, ill-defined lesions in the bilateral cerebral, basal ganglia, cerebellum, and brainstem. MRI spine showed enhancing lesions in spinal cord and cauda equina nerve roots. CSF analysis showed 13 nucleated cells (100% lymphocytes) and normal protein. Oligoclonal bands and IgG index were not done. Flow cytometry and cytology were unrevealing. Brain biopsy showed non-specific chronic inflammatory changes with macrophages/monocytes; findings were not consistent with demyelination or CNS lymphoma and broad infectious testing was negative. MRI brain 3 months later showed significantly decreased enhancing lesions, but interval hemorrhage of multiple supratentorial and infratentorial lesions. Autopsy 4 months later showed diffuse inflammatory infiltrates predominantly composed of macrophages and T-cells, with organizing necrosis and perivascular lymphocytic infiltration.

Patient 41: Patient with history of colon cancer status post resection and chemotherapy 10 years prior, atrial flutter on Xarelto, and cerebrovascular accident 1 year prior. He presented with transient loss of vision in the superior visual field on the left eye. Fundoscopic exam revealed optic disc edema in the left eye. C-reactive protein was 20 mg/L and sedimentation rate was 77 mM/Hr. MRI brain and orbit showed no definite orbital abnormality, though limited by motion artifact. His temporal artery biopsy was negative.

Patient 42: Patient failed vision test while undergoing testing for pilot license, prompting ophthalmological evaluation. OCT with symmetric severe thinning.

Patient 43: Patient with complex medical history including heart failure with reduced ejection fraction, end stage renal disease on hemodialysis, type 2 diabetes, paroxysmal atrial fibrillation with cerebrovascular accidents and severe vascular dementia/neurocognitive decline. He presented with worsening altered mental status. CSF analysis with 0 nucleated cells and normal protein. Oligoclonal bands and IgG index were not done. MRI brain initially showed multiple scattered foci of restricted diffusion and on repeat 3 weeks later showed several new foci of restricted diffusion.

Patient 44: Patient presented with progressive bilateral weakness and numbness, starting in the feet, then hands and generalized. MRI brain and spine were normal. EMG/NCS showed severe sensorimotor polyneuropathy with demyelinating features. CSF analysis showed 3 nucleated cells and normal protein, with negative oligoclonal bands and normal IgG index.

Patient 45: Patient presented with progressive blurry vision over 1 month associated with redness, pruritus and watery drainage. He has history of HIV (CD4 counts at time of evaluation <200 and he was not on antiretroviral therapy). Exam with retinal hemorrhages bilaterally and suspicious granular area inferotemporally on the left with chorioretinal scars. CSF analysis was not done.

Patient 46: Patient initially presented with headaches, photophobia and phonophobia, and during admission developed confusion and had fevers for 11 days. CSF analysis initially showed 192 nucleated cells (72% lymphocytes, 15% neutrophils) and on repeat 1.5 weeks later showed 704 nucleated cells (52% lymphocytes, 44% neutrophils). Oligoclonal bands and IgG index were not done. MRI brain initially with patchy areas of subtle leptomeningeal enhancement throughout the bilateral cerebral hemispheres, with subtle areas of associated parenchymal signal abnormality and on repeat 1.5 weeks showed additional leptomeningeal enhancement in lower brainstem as well as new ventriculitis. Outpatient MRI 7 months later showed resolution of the enhancements and increased prominence of ventricular size.

Patient 47: Patient presented with a variety of neurological symptoms including severe gait impairment, inability to use hands properly, fatigue, and brain fog following COVID-19 vaccine. On exam, she had difficulty with activation of some of the muscle groups. With extensive coaching, however, she was able to do so with full strength. She displayed an astasia-abasia gait. MRI brain with non-specific white matter changes. MRI cervical spine with punctuate left sided hyperintense lesion at C5 where there is prominent disc disease. CSF analysis with normal nucleated cells and protein. Oligoclonal bands and IgG index were not done. Electromyography/nerve conduction study was normal.

Patient 48: Patient with history of hemoglobin sickle C disease, bilateral pulmonary emboli on Xarelto and migraines who presented with intermittent, painless left eye vision loss with relative pupillary afferent defect. MRI brain and orbit showed partially empty sella and flattening of the posterior globe bilaterally. Fundoscopic exam with bilateral optic nerve head edema. Opening pressure was 21.5 mmHg. CSF analysis with normal nucleated cells and protein. Oligoclonal bands and IgG index were not performed. She was started on Diamox for presumed idiopathic intracranial hypertension. Two months later she presented with altered mental status and was found to have multifocal punctuate infracts and microhemorrhages thought due to vaso-occlusive crisis. She improved with red blood cell exchange.

Patient 49: Patient had an episode of headache and transient loss of peripheral vision out of her left eye which lasted for a few minutes and resolved. After that episode, she had intermittent headaches but no loss of vision. MRI brain done 2 months later showed one periventricular lesion and two small non-specific subcortical white matter lesions. MRI spine negative. CSF analysis was negative for oligoclonal bands.

Patient 50: Patient has history of recurrent Bell’s palsy who was found to have visual field defect on routine follow up with ophthalmology. MRI orbit negative and OCT with normal RNFL over two year period.

Patient 51: Patient with history of systemic lupus erythematous, antiphospholipid syndrome and patent foramen ovale who had neurological events more consistent with ischemic events. OCT showed decrement in the retinal nerve fiber layer in the left eye, congruous with an ipsilateral inferior macular/inner retinal deficit, which is consistent with an ischemic optic neuropathy of the left eye.

**Supplemental tables**

eTable 1. List of typical and atypical features assessed on MRI.

| MRI | | Description |
| --- | --- | --- |
| Brain | Typical | Multiple ill-defined T2 hyperintense lesions in supratentorial and often infratentorial white matter |
|  |  | Deep gray matter involvement |
|  |  | Ill-defined T2 hyperintensity involving pons, middle cerebellar peduncle or medulla |
|  |  | Cortical lesions with or without lesional enhancement |
|  |  | Meningeal enhancement |
|  | Atypical | Dawson’s fingers |
|  |  | Linear lesion along corticospinal tract or medulla |
|  |  | Thin periependymal lining surrounding ventricular system or diencephalic lesions surrounding third ventricle or area postrema lesions |
|  |  | Ovoid, ring or open-ring enhancement |
|  |  | Pencil thin enhancement of ependymal surface of lateral ventricles |
|  |  | Patchy, cloud like enhancement of brain lesions |
| Orbit | Typical | Bilateral involvement |
|  |  | >1/2 length of optic nerve |
|  |  | Perineural optic sheath enhancement |
|  |  | Flattening of posterior globe |
|  | Atypical | Chiasmal or optic tract involvement |
| Spine | Typical | Longitudinally extensive |
|  |  | Central cord lesion or H-sign |
|  |  | Conus lesion |
|  | Atypical | Multiple focal short segment skip lesions (without longitudinally extensive lesion) |
|  |  | Posterior and involving only portion of the cross-sectional area of cord |
|  |  | Bright spotty lesions |

eTable 2. Comparison of the MRI manifestations of true positive cases in children and adults based on imaging location.

| Image location | Typical/atypical | Specific imaging feature | Total cohort | Children | Adult | P-value |
| --- | --- | --- | --- | --- | --- | --- |
| Orbit | Typical | Bilateral involvement | 31/137 (22.6%) | 24/84 (28.6%) | 7/53 (13.2%) | 0.036 |
|  |  | >1/2 length of optic nerve | 68/137 (49.6%) | 37/84 (44.0%) | 31/53 (58.5%) | 0.099 |
|  |  | Perineural optic sheath enhancement | 56/136 (41.2%) | 31/83 (37.3%) | 23/53 (43.4%) | 0.482 |
|  |  | Flattening of posterior globe | 35/137 (25.5%) | 28/84 (33.3%) | 7/53 (13.2%) | 0.009 |
|  | Atypical | Chiasmal or optic tract involvement | 8/137 (5.8%) | 4/84 (4.8%) | 4/53 (7.5%) | 0.498 |
| Spine | Typical | Longitudinal extensive | 33/113 (29.2%) | 23/68 (33.8%) | 10/45 (22.2%) | 0.184 |
|  |  | Central cord lesion or H-sign | 34/112 (30.4%) | 26/67 (38.8%) | 8/45 (17.8%) | 0.017 |
|  |  | Conus lesion | 23/109 (21.1%) | 19/68 (27.9%) | 4/41 (9.8%) | 0.024 |
|  | Atypical | Multiple focal short segment skip lesions (without longitudinally extensive lesion) | 8/113 (7.1%) | 4/68 (5.9%) | 4/45 (8.9%) | 0.542 |
|  |  | Posterior and involving only portion of the cross-sectional area of cord | 9/112 (8.0%) | 4/67 (6.0%) | 5/45 (11.1%) | 0.326 |
|  |  | Bright spotty lesions | 3/113 (2.7%) | 3/68 (4.4%) | 0/45 (0.0%) | 0.275 |
| Brain | Typical | Multiple ill-defined T2 hyperintense lesions in supratentorial and often infratentorial white matter | 53/150 (35.3%) | 43/86 (50.0%) | 10/64 (15.6%) | <0.001 |
|  |  | Deep gray matter involvement | 46/150 (48.8%) | 42/86 (48.8%) | 4/64 (7.8%) | <0.001 |
|  |  | Ill-defined T2 hyperintensity involving pons, middle cerebellar peduncle or medulla | 44/150 (29.3%) | 32/86 (37.2%) | 12/64 (18.8%) | 0.014 |
|  |  | Cortical lesions with or without lesional enhancement | 48/150 (32.0%) | 43/86 (50.0%) | 5/64 (7.8%) | <0.001 |
|  |  | Meningeal enhancement | 24/148 (16.2%) | 22/84 (26.2%) | 2/64 (3.1%) | <0.001 |
|  | Atypical | Dawson’s fingers | 2/150 (1.3%) | 1/86 (1.2%) | 1/64 (1.6%) | 0.833 |
|  |  | Linear lesion along corticospinal tract or medulla | 9/150 (9.3%) | 8/86 (9.3%) | 1/64 (1.6%) | 0.048 |
|  |  | Thin periependymal lining surrounding ventricular system or diencephalic lesions surrounding third ventricle or area postrema lesions | 6/150 (4.0%) | 6/86 (7.0%) | 0/64 (0.0%) | 0.038 |
|  |  | Ovoid, ring or open-ring enhancement | 4/148 (2.7%) | 3/84 (3.6%) | 1/64 (1.6%) | 0.455 |
|  |  | Pencil thin enhancement of ependymal surface of lateral ventricles | 2/148 (1.4%) | 2/84 (2.4%) | 0/64 (0.0%) | 0.506 |
|  |  | Patchy, cloud like enhancement of brain lesions | 26/148 (17.6%) | 16/84 (19.0%) | 10/64 (15.6%) | 0.588 |

eTable 3. Comparison of the MRI manifestations of true positive cases in children and adults based on clinical presentations.

| Clinical presentation | Typical/atypical | Specific imaging feature | Total cohort | Children | Adult | P-value |
| --- | --- | --- | --- | --- | --- | --- |
| Optic neuritis | Typical | Bilateral involvement | 25/75 (33.3%) | 18/32 (56.3%) | 7/43 (16.3%) | <0.001 |
|  |  | >1/2 length of optic nerve | 57/75 (76.0%) | 28/32 (87.5%) | 29/43 (67.4%) | 0.044 |
|  |  | Perineural optic sheath enhancement | 46/75 (61.3%) | 25/32 (78.1%) | 21/43 (48.9%) | 0.010 |
|  |  | Flattening of posterior globe | 27/75 (36.0%) | 19/32 (59.4%) | 8/43 (18.6%) | <0.001 |
|  | Atypical | Chiasmal or optic tract involvement | 6/75 (8.0%) | 2/32 (6.3%) | 4/43 (9.3%) | 0.630 |
| Myelitis | Typical | Longitudinal extensive | 14/23 (60.9%) | 6/7 (85.7%) | 8/16 (50.0%) | 0.176 |
|  |  | Central cord lesion or H-sign | 12/23 (56.5%) | 6/7 (85.7%) | 7/16 (43.8%) | 0.089 |
|  |  | Conus lesion | 8/21 (38.1%) | 5/7 (71.4%) | 3/14 (21.4%) | 0.056 |
|  | Atypical | Multiple focal short segment skip lesions (without longitudinally extensive lesion) | 3/23 (13.0%) | 0/7 (0.0%) | 3/16 (18.8%) | 0.526 |
|  |  | Posterior and involving only portion of the cross-sectional area of cord | 4/23 (17.4%) | 0/7 (0.0%) | 4/16 (25.0%) | 0.273 |
|  |  | Bright spotty lesions | 0/23 (0.0%) | 0/7 (0.0%) | 0/16 (0.0%) | 1.000 |
| Brain, brainstem, or cerebral syndrome | Typical | Multiple ill-defined T2 hyperintense lesions in supratentorial and often infratentorial white matter | 43/56 (76.8%) | 38/48 (79.2%) | 5/8 (62.5%) | 0.370 |
|  |  | Deep gray matter involvement | 40/56 (71.4%) | 35/48 (72.9%) | 5/8 (62.5%) | 0.676 |
|  |  | Ill-defined T2 hyperintensity involving pons, middle cerebellar peduncle or medulla | 34/56 (60.7%) | 27/48 (56.3%) | 7/8 (87.5%) | 0.130 |
|  |  | Cortical lesions with or without lesional enhancement | 42/56 (75.0%) | 37/48 (77.1%) | 5/8 (62.5%) | 0.458 |
|  |  | Meningeal enhancement | 21/54 (38.9%) | 19/46 (41.3%) | 2/8 (25.0%) | 0.461 |
|  | Atypical | Dawson’s fingers | 1/56 (1.8%) | 1/48 (2.1%) | 0/8 (0.0%) | 1.000 |
|  |  | Linear lesion along corticospinal tract or medulla | 7/56 (12.5%) | 7/48 (14.6%) | 0/8 (0.0%) | 0.577 |
|  |  | Thin periependymal lining surrounding ventricular system or diencephalic lesions surrounding third ventricle or area postrema lesions | 4/56 (7.1%) | 4/48 (8.3%) | 0/8 (0.0%) | 1.000 |
|  |  | Ovoid, ring or open-ring enhancement | 4/54 (7.4%) | 3/46 (6.5%) | 1/8 (12.5%) | 0.484 |
|  |  | Pencil thin enhancement of ependymal surface of lateral ventricles | 2/54 (3.7%) | 2/46 (4.3%) | 0/8 (0.0%) | 1.000 |
|  |  | Patchy, cloud like enhancement of brain lesions | 20/54 (37.0%) | 14/46 (30.4%) | 6/8 (75.0%) | 0.041 |
